# A QbD-Navigated Approach to the Development and Evaluation of Etodolac–Phospholipid Complex Containing Polymeric Films for Improved Anti-Inflammatory Effect

**DOI:** 10.3390/polym16172517

**Published:** 2024-09-04

**Authors:** Jangjeet Karan Singh, Simran Kaur, Balakumar Chandrasekaran, Gurpreet Kaur, Balraj Saini, Rajwinder Kaur, Pragati Silakari, Narinderpal Kaur, Pallavi Bassi

**Affiliations:** 1 Chitkara College of Pharmacy, Chitkara University, Rajpura 140401, Punjab, India; jangjeet21004.ccp@chitkara.edu.in (J.K.S.); simran22010.ccp@chitkara.edu.in (S.K.); balraj.saini@chitkara.edu.in (B.S.); rajwinder.kaur@chitkara.edu.in (R.K.); pragati.silakari@chitkara.edu.in (P.S.); 2 Faculty of Pharmacy, Philadelphia University, P.O. Box 1, Amman 19392, Jordan; 3 Department of Pharmaceutical Sciences and Drug Research, Punjabi University, Patiala 147002, Punjab, India; kaurgpt@gmail.com; 4 Chitkara University School of Pharmacy, Chitkara University, Baddi 174103, Himachal Pradesh, India; narinder.sonia@gmail.com

**Keywords:** QbD, phospholipid complex, transdermal, health, research, response surface methodology

## Abstract

The current study focuses on development of phospholipid complex-loaded films of etodolac for enhanced transdermal permeation and anti-inflammatory effect. An etodolac–phospholipid complex was developed using the solvent evaporation method and was characterized by DSC, XRD, FTIR, and ^1^H-NMR studies. The formation of the complex led to conversion of a crystalline drug to an amorphous form. A stoichiometric ratio of 1:1 (drug–phospholipid) was selected as the optimized ratio. Further, the developed complex was incorporated into films and systematic optimization using a central composite design was carried out using a response surface methodological approach. The desirable design space based on minimum contact angle and maximum tensile strength was selected, while the water vapour transmission rate and swelling index were set within limits. The results for swelling index, contact angle, tensile strength, and water vapour transmission rate were 60.14 ± 1.01%, 31.6 ± 0.03, 2.44 ± 0.39 kg/cm^2^, and 15.38 g/hm^2^, respectively. These values exhibited a good correlation with the model-predicted values. The optimized formulation exhibited improved diffusion and permeation across skin. In vivo studies revealed enhanced anti-inflammatory potential of the developed films in comparison to the un-complexed drug. Hence, the study demonstrated that etodolac–phospholipid complex-loaded films improve the transdermal permeation and provided enhanced anti-inflammatory effect.

## 1. Introduction

Osteoarthritis (OA) represents a complex and persistent degenerative disorder that affects the joints and is characterized by the progressive degeneration of cartilage. The condition can cause pain, stiffness, and limited mobility. It is highly prevalent in society and is one of the leading causes of disability. According to the WHO, about 528 million people are affected by OA worldwide, with 73% of individuals above the age of 55 [1]. This prevalence is expected to rise further due to increasing aging demography and the obesity epidemic. 

Non-steroidal anti-inflammatory drugs (NSAIDs) are the most-commonly prescribed category of drugs for symptomatic treatment of OA. Etodolac (ETO), a pyranocarboxylic acid derivative, is an NSAID, FDA-approved since 1991. It is used in the management of arthritis, exhibiting anti-inflammatory, analgesic, and antipyretic activities. However, it possesses limited solubility and has a half-life of about 7 h, which necessitates frequent dosing. Also, prolonged use of ETO may result in severe GI disturbances such as intestinal bleeding, ulcers, etc. This necessitates the use of alternative strategies like different routes of administration. Transdermal drug delivery systems, especially films, are a convenient, non-messy and patient-friendly avenue in this regard. In addition, they offer better adhesion and permeation at the target site. 

Various techniques have been explored to enhance the biopharmaceutical profile of drugs. These include the hot melt extrusion approach, microemulsion systems, formulation of niosomal gel [2], nanosuspension-based gel [3], cubosomes [4], and self-emulsifying drug delivery system (SEDDS) or 3D-printed materials [5,6]. However, these techniques have inherent limitations such as low payload, drug leakage upon storage, and poor stability. One effective approach to address these challenges involves the interaction of drugs with phospholipids and the development of a drug–phospholipid complex. The formation of covalent or non-covalent bonds with phospholipids may enhance the permeation, entrapment efficiency, and stability. Additionally, the phospholipids are biocompatible in nature and can form a bilayer structure which mimics the biological cell membrane [7,8]. 

Systematic optimization of various active and inactive ingredients is imperative for the development of a robust drug formulation in order to attain the intended product quality. The traditional method of modifying a single variable at a time is laborious, prone to errors, and time-consuming, and frequently results in solutions and products that are only moderately effective and have limited robustness. On the other hand, the Quality by Design (QbD) approach is a risk-based, scientific, and systematic approach utilizing experimental designs that necessitate only minimal experimentation in order to produce the most effective formulation. QbD provides exceptional advantages, including time, effort, and financial savings, apart from providing clear comprehension of the involved products and processes. Recent research has focused extensively on the application of QbD for the development of consistent and robust drug delivery systems and therapeutic efficacy [9].

In the present investigation, a phospholipid complex of etodolac was prepared with the aim to enhance transdermal permeation. The complex was thoroughly characterized, encompassing percentage yield, solubility, and surface morphology. Further, the complex was loaded in films, and this formulation was comprehensively evaluated in vitro and in vivo.

## 2. Materials and Methods

### 2.1. Materials

Etodolac (ETO) and carrageenan were obtained from Yarrow Pharma Pvt. Ltd., Mumbai, India. Soy lecithin phospholipid (PL) was purchased from Himedia laboratories, Thane, India. Dichloromethane and methanol were purchased from Rankem Chemicals, Thane, Maharashtra, India. Polyvinyl alcohol, PEG 400, and *n*-cctanol were purchased from Loba Chemie Pvt. Ltd., Mumbai, India. Methyl cellulose was purchased from Thomas Baker (Chemicals) Pvt. Ltd., Mumbai, India. All the chemicals were of analytical grade and were used as procured.

### 2.2. Methods

#### 2.2.1. Molecular Docking Studies

The computer-aided molecular docking studies were executed between Etodolac (ETO) and the phosphatidylcholine transfer protein (i.e., nearby analog of PL in the humanoid physique) to analyze the possible interaction of drug and phospholipid [10]. The CHARMm-based docking program CDOCKER of the Discovery Studio Client v20.1.0.19295 software was used to perform the molecular docking simulation [11]. Using the Discovery Studio Client v20.1.0.19295 workspace, the test molecule was drawn and processed. Next, energy was minimized using the “Prepare Ligands” tool at pH 7.4. The Protein Data Bank [12] provided the X-ray crystallographic structure of the phosphatidylcholine transfer protein (PDB ID: 1LN1), which was subsequently optimized for docking analysis [13]. As part of the optimization technique, hydrogen atoms were added, water molecules were removed, bond orders were completed, and hydrogen bonds were assigned. With the use of the CDOCKER program’s CHARMm-based docking tool, the test drug was docked into the protein active site. The calculated binding energy of the protein hit was found to be a negative value, suggesting its stable interaction energy [14].

#### 2.2.2. Preparation of Etodolac–Phospholipid Complex (ETO–PLC)

Etodolac–phospholipid (ETO–PLC) complex was prepared by the solvent evaporation method. Briefly, equimolar solutions of ETO and PL were separately prepared in dichloromethane. The PL solution was then gradually added to the ETO solution at different ratios under continuous stirring. The resultant solution was then refluxed at 30 °C for 4 h and the solvent was removed from the reaction mixture under reduced pressure using a Rota evaporator (Perfit, India, model no. VP 1000) to get a solid product. The prepared complex was kept in a desiccator until further use [7]. 

#### 2.2.3. Determination of Optimum Stoichiometric Ratio for Complex Formation 

A Job plot (method of continuous variation) was used to determine the optimum complexation ratio for ETO and PL [15]. Equimolar solutions of ETO and PL were taken in different ratios. Additionally, ETO dilutions in methanol served as a control. The absorbance of ETO control and its corresponding complex was measured at 273 nm, using a UV-Visible spectrophotometer (Systronics 2205, Gujarat, India), and the difference in the absorbance (ΔA) was determined. ΔA vs. the corresponding mole fraction of ETO was plotted to obtain the Job plot. The ratio pertaining to maximum ΔA was selected as the optimized ratio for the preparation of ETO–PLC [16].

#### 2.2.4. Characterization of the Complex

##### Estimation of Gibbs Free Energy

A thermodynamic analysis of the interaction between PL and ETO with solvent at the interface was carried out. The effect of the addition of PL on ETO solubilization was analyzed using Gibbs free energy, employing Equation (1):

(1) $$\Delta G\;=-2.303\mathrm{RT}\;\mathrm{Log}\;\frac{S}{S_{O}}\;$$

where ΔG is Gibbs free energy (kJ/mol), R is the universal gas constant (8.314 J/K mol), T is the absolute temperature (Kelvin), and *S_o_* and *S* is the solubility of ETO and ETO–PLC (mg/mL), respectively [17]. The extent of increase in the solubility of the complex vis-à-vis pure drug was deduced from the values of Gibbs free energy. Further, the equilibrium solubility of the optimized complex was determined in different solvents, namely, distilled water, phosphate buffer pH 7.4, and 0.1 N HCl using a previously reported method [18,19].

##### Percentage Yield (% Yield)

Percentage yield refers to the percent amount of actual yield to that of theoretical yield. It was calculated using Equation (2):

(2) $$\%\;yield=\frac{Actual\;yield\;}{Theoretical\;yield\;}\times 100$$

#### 2.2.5. Fourier Transform Infrared Spectroscopy (FTIR) Studies

Samples of ETO, PL, and ETO–PLC were analyzed using FTIR in the range of 4000–400 per cm at a resolution of 2/cm. Different spectra were assessed for peaks related to different functional groups and peak shifts of representative spectra were studied in comparison to that of FTIR spectra of ETO.

#### 2.2.6. X-ray Diffraction Studies (XRD)

The XRD patterns of ETO and ETO–PLC were recorded using an X’Pert Pro^®^ diffractometer, PAN analyticals, The Netherlands. The X-ray source consisted of a copper tube (wavelength 1.54 Å). The operating conditions were 45 kV, 40 mA, and a scanning range of 5° to 50°.

#### 2.2.7. Differential Scanning Calorimetry (DSC)

The thermal behavior of ETO and ETO–PLC were recorded using SETRAM SETLINE DSC+ Caluire, France at a heating rate of 0.01 to 100 (°C/min). 

#### 2.2.8. Scanning Electron Microscopy (SEM) Studies

The surface morphology and shape of the ETO–PLC were visualized under a scanning electron microscope (JSM-6510LV). Before visualization, the samples were mounted onto slabs using double-sided carbon tape and vacuum-coated with gold–palladium film (thickness 2 nm) using a sputter coater to render them electrically conductive. The microscope was operated at an acceleration voltage of 5 kV and obtained micrographs were visualized at a magnification of 1000× 10,000× and 15,000×. 

#### 2.2.9. ^1^H-NMR Studies

^1^H-NMR spectroscopy for ETO and optimized ETO–PLC was carried out using a Bruker Avance Neo, Rheinstetten, Germany, 500 MHz NMR spectrometer in CDCl_3_. All the respective spectra were analyzed for any chemical shifts of peaks to validate complex formation [7].

#### 2.2.10. Mathematical Modelling and Optimization of Blank Films Using the QbD Approach

##### Identifying the Quality Target Product Profile (QTPP)

A Quality by Design approach was used for the development of blank films. All the quality targets were set so as to improve the overall performance of the formulation. The QTPP considered included the type of formulation, route of administration, and stability.

##### Determination of Critical Quality Attributes (CQAs)

The prominent factors affecting the performance of film formulation were identified. These included the swelling index, contact angle, tensile strength, and water vapour transmission rate (WVTR). These factors affect the water uptake, contact with application site, mechanical strength of the dosage form, and the moisture loss from the application site, respectively.

##### Risk Assessment

Critical material attributes (CMAs) and critical process parameters (CPPs) were identified through a risk assessment matrix. An Ishikawa fishbone diagram was constructed to identify various CPPs and CMAs that influence the quality of the formulation. After the risk assessment, two factors i.e., polymer concentration and plasticizer concentration, were identified as the important factors affecting the formulation development.

##### Experimental Design

JMP^®^ student subscription 17.1.0 software was employed for mathematical modelling and optimization. Systematic optimization of films was carried out through a response surface methodological approach. A central composite design with two independent factors (X) namely PVA (polymer) concentration (X1) and PEG400 (plasticizer) concentration (X2), consisting of 10 runs with two replicate center points was employed (Table 1). The independent factors were chosen to be studied at 3 levels (−1, 0, +1), namely, low (−1), medium (0), and high (+1), as shown in Table 1. Response variables, included % swelling index (Y1), contact angle (Y2), tensile strength (Y3), and water vapor transmission rate (Y4).

#### 2.2.11. Preparation of Transdermal Films

The transdermal films were prepared by the solvent casting method. The weighed quantities of polymers (methylcellulose, polyvinyl alcohol) were dissolved in distilled water. Thereafter, PEG 400 was added to the polymer mixture under continuous stirring. The prepared solution was then sonicated to remove any entrapped bubbles. The solution was cast in the polypropylene petri plates and was dried in an oven at 60 °C. The dried films were removed from the plates and stored in a desiccator. Drug-loaded films were prepared in similar manner, where a complex analogous to 300 mg of ETO was added to a polymeric solution.

#### 2.2.12. Evaluation and Characterization of Transdermal Film

##### Thickness

The thickness of the films was measured in triplicate from randomly selected portions of the film using vernier calipers. The mean thickness was determined.

##### Weight Variation

The films were cut to an area of 1 cm^2^ and weighed on digital balance (Citizen CY 220, Mumbai, India). All measurements were carried out in triplicate.

##### Folding Endurance

The folding endurance test was performed by manually folding the films. The films which could undergo folding more than 300 times without breaking were selected for further studies.

##### Surface pH Determination

Films with a surface area 1 cm^2^ were cut and placed in glass petri plates containing distilled water and were allowed to swell. After 1 h, the swollen film was removed, and its pH was recorded using a pH meter [20].

##### Swelling Index (SI)

The SI was determined by placing the films in freshly prepared phosphate buffer pH 7.4 after recording their initial weights. The film weight was recorded at predetermined time intervals [21]. The % SI of the film was calculated by the following formula.

(3) $$\mathrm{Swelling}\;\mathrm{index}\;\left(\%\right)=\frac{\mathrm{Final}\;\mathrm{weight}\;\mathrm{of}\;\mathrm{film}-\mathrm{Initital}\;\mathrm{weight}\;\mathrm{of}\;\mathrm{film}\;}{\mathrm{Initial}\;\mathrm{weight}\;\mathrm{of}\;\mathrm{film}\;}\times 100$$

##### Tensile Strength (TS)

Mechanical properties play an important role in the stability and drug release from the film. Tensile strength was determined using a texture analyzer (TA.XT plus C, Stable Microsystems, Surrey, UK). A 2 × 2 cm patch was placed between two grips of the texture analyzer. The force required to break the film was used as a measure of tensile strength. The tensile strength was calculated by the following formula [22].

(4) $$\mathrm{Tensile}\;\mathrm{strength}\;\left(\mathrm{kg}/{\mathrm{cm}}^{2}\right)=\frac{\mathrm{Breaking}\;\mathrm{force}\;\left(\mathrm{kg}\right)}{\mathrm{Cross}\;\mathrm{Sec}\mathrm{tional}\;\mathrm{area}\;\mathrm{of}\;\mathrm{samle}\left({\mathrm{cm}}^{2}\right)}$$

##### Contact Angle (CA)

The CA provides information about the wetting properties of the film. CA was determined by gently placing a drop of phosphate buffer pH 7.4 on the surface of the film with the help of a micropipette from a distance of 1 cm from the film surface. The distance was fixed to ensure uniformity between measurements. ImageJ software (version 1.8.0) was used to estimate the angle between the tangent line of the drop and the film surface.

##### Determination of Water Vapor Transmission Rate (WVTR)

The WVTR depicts the rate of moisture exchange through the films and indirectly provides insight into hydrophilic character and barrier properties of the developed film. For the estimation of WVTR, film was placed and sealed on the top of a 10 mL beaker which was filled with 8.9 mL of distilled water, giving an air gap of 1.1 cm from the film underside. The system was kept at room temperature (25 ± 2 °C, 40 ± 2% RH) for 2 h for equilibration, followed by measurement of WVTR (g/hm^2^) using a VapoMeter (Delfin Technologies Ltd., Kuopio, Finland) [23]. The closed chamber design of the VapoMeter helped to nullify the influence of external air flow so as to enable measurements under normal room conditions. 

#### 2.2.13. Selection of Optimized Film Using Point Prediction Method 

A numerical optimization technique using a desirability approach was employed to identify the desired design space. Numerical optimization was carried out after setting different limits. These included the minimum contact angle and maximum TS. The WVTR and SI were set with maximum limits of 16.5 g/hm^2^ and 70%, respectively. From the obtained design space, one optimized composition was selected and experimentally validated. The model was validated by correlating the observed and the model-predicted values.

#### 2.2.14. In Vitro Diffusion Studies

In vitro dissolution studies were carried out using Franz diffusion cells. The samples were placed over the diffusion membrane (6000−8000 Da) in the donor compartment. The receptor chamber containing phosphate buffer pH 7.4 was stirred continuously using a magnetic bead. The system was maintained at 37 ± 0.5 °C. Samples (2 mL) were withdrawn from the sampling port at predetermined intervals and replenished with an equal volume of fresh dissolution media. Drug content was determined by analyzing the samples using a UV-visible spectrophotometer and measuring their absorbance at λmax 273 nm. The % drug release calculated was plotted as a function of time. Further release data were fitted into different kinetic equations to estimate the rate and mechanism of drug release from film formulation.

#### 2.2.15. Ex Vivo Permeation Studies 

A Franz diffusion cell was used for this study. Hairless rat skin was taken and was clamped (diffusion area 2.5 cm^2^) between the donor and receptor compartment. The receptor chamber was filled with phosphate buffer pH 7.4, maintained at a temperature of 37 °C ± 1 °C, and kept under a stirring condition (100 rpm). Film formulation was gently placed on the skin. The samples were withdrawn at predetermined time intervals and replenished with an equal volume of fresh media. The withdrawn samples were analyzed using a UV spectrophotometer after filtration through a 0.45 µm membrane filter. The cumulative drug permeated amount per unit area (µg/cm^2^) and flux were calculated [24]. 

#### 2.2.16. In Vivo Animal Studies/Paw Edema Method

Inbred adult male Wistar rats weighing 220 ± 20 g and having free access to standard feed and water ad libitum were employed in the present study after approval from the institutional animal ethical committee. All animals were kept according to the Guide for the Care and Use of Laboratory Animals. The animals were housed in the departmental animal house and were exposed to a cycle of 12 h light and 12 h dark. Twenty-four Wistar rats were randomly selected and divided into groups, namely, the control group (no treatment), the disease control group (carrageenan-induced inflammation), treatment group I (carrageenan-induced treated with ETO films), and treatment group II (carrageenan-induced treated with ETO–PLC films). Carrageenan (1% *w*/*v*) was used to induce inflammation in the left paw of the disease control and treatment groups. Films were applied to the induced inflammation region after noting the initial paw volume. The paw volume was measured at regular time intervals using a digital plethysmometer [25].

#### 2.2.17. Statistical Analysis

All the experimental work was conducted in triplicate, and results are reported as mean ± SD. A *t*-test was employed where applicable to judge the statistical significance at a 95% confidence interval.

## 3. Results and Discussion

### 3.1. Molecular Docking Studies

**Figure 1:**
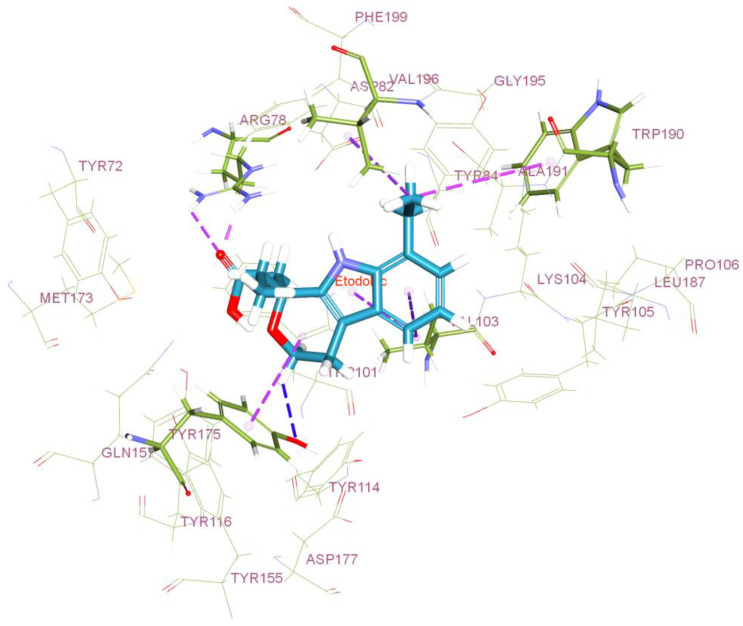
Three-dimensional interactions of ETO with phosphatidylcholine transfer protein: The hydrogen bonds are indicated with blue dashed lines, and the aromatic interactions are in purple dashed lines.

Exploring the drug–ligand docking analysis, potential site(s) of interaction between the drug and PL analog (i.e., phosphatidylcholine transfer protein) were identified. Mechanistically, the research showed that the substance and protein formed weak intermolecular hydrogen bonding connections. The drug’s docking patterns of hydrogen bonds between the molecule’s free hydroxyl groups and the protein’s terminal quaternary amine group, as well as aromatic contacts in the hydrophobic region of the protein, are depicted in Figure 1. This is due to the drug’s slight decrease in activation energy following complexation with proteins through the formation of H-bonds, which ultimately results in the minimization of total free energy required to achieve thermodynamically stable confirmation. In general, the molecular docking experiments supported the drug’s ability to form a complex with the phosphatidylcholine transfer protein, most likely because the protein’s physiochemical characteristics are structurally comparable to those of phospholipids [10].

### 3.2. Job Plot, Solubility, and Gibb’s Free Energy

**Figure 2:**
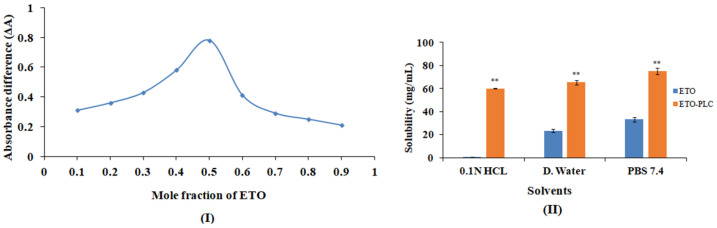
(**I**) Job plot depicting absorbance difference with respect to mole fraction of ETO. (**II**) Solubility of ETO and the ETO–PLO complex in different solvents. Solubilities are represented as mean ± SD (*n* = 3). ** *p* < 0.05 as compared to ETO at the same pH value.

**Figure 3:**
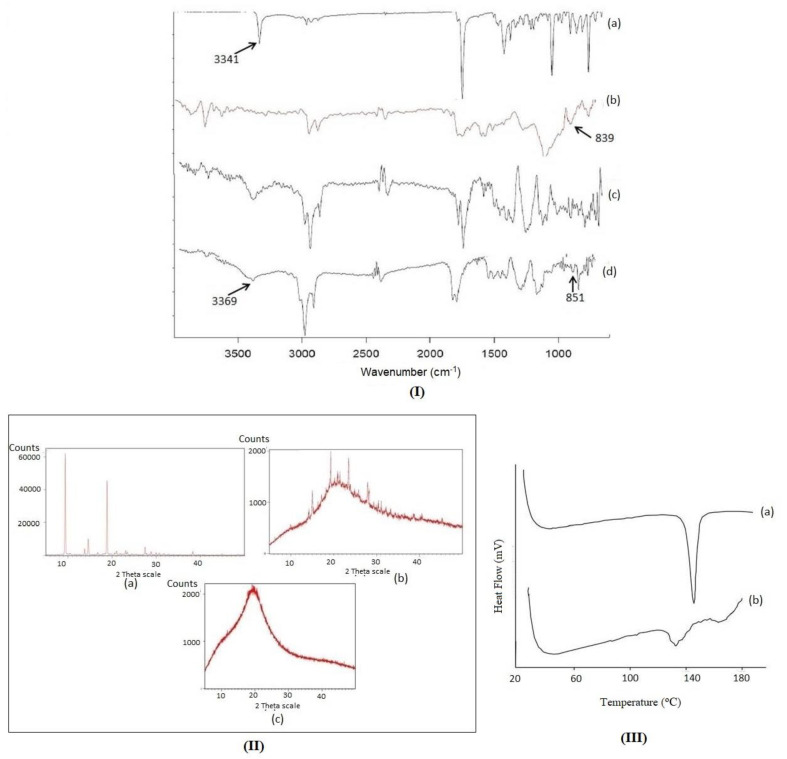
(**I**) FTIR spectra of ETO (**a**), PL (**b**), ETO–PLC 4:6 ratio (**c**), ETO–PLC 1:1 Ratio (**d**).; (**II**) XRD diffractograms of ETO (**a**), ETO–PLC 4:6 ratio (**b**) and ETO–PLC 1:1 ratio (**c**); (**III**) DSC thermograph of ETO (**a**) and ETO -PLC (1:1) (**b**)

The Job plot was constructed by plotting ΔA against the corresponding mole fraction. The maximum value of ΔA was obtained at a stoichiometric ratio of 1:1 (ETO–PL). The ∆G value for all the stoichiometric ratios was negative, indicating the spontaneity of the reaction. The negative values also indicate that the complexation process led to the release of energy as a result of the development of Van der Waals forces of attraction and/or electrostatic interaction between ETO and PL [26]. The value of ∆G decreased as the PL content increased, indicating that the complex formation became more favorable at higher concentrations of PL, and was maximum at a 1:1 ratio (−7.03 KJ/mol). Beyond this ratio, ∆G values showed an increase, signifying that the maximum solubility was attained at a 1:1 ratio. Hence, a 1:1 complex was selected as the optimized complex. The solubility of the ETO and optimized ETO–PLC was also carried out in different solvents and is depicted in Figure 2. Etodolac exhibits pH-dependent solubility, demonstrating minimum solubility in 0.1 N HCL, and a significantly high solubility in water and PBS 7.4. This is because etodolac is a weakly acidic drug with a pKa of 4.65. The solubility shows a marked increase when pH rises above the pKa value [27]. The solubility of ETO demonstrated a significant increase with complex formation in all the media. Furthermore, a pH-independent solubility was observed after complex formation (Figure 2). Drug phospholipids have been reported to withstand alterations in pH [8]. The enhancement in solubility could be ascribed to the amorphous nature of the ETO–PLC, which overrides the lattice energy required for solubilization of crystalline compounds. The amorphous nature of ETO–PLC was evident in XRD studies (Figure 3). Additionally, PL has surface active properties and is amphiphilic. This results in self-assembly in water to form micelles that facilitate the solubilization of the drug [28,29].

### 3.3. Percentage Yield

The percentage yield of the optimized ETO–PLC (1:1) was found to be 86.72 ± 1.21% by Equation (2). This suggests that complexation was relatively efficient and that the conditions under which the reaction was carried out were appropriate.

### 3.4. FTIR Studies

Figure 3(I) illustrates the FTIR spectra of ETO, PL, and ETO–PLC at a ratio of 4:6 and ETO–PLC at a 1:1 ratio. The FTIR spectra of ETO showed characteristic peaks at 3341 cm^−1^ (OH group of carboxylate mode), 2971 cm^−1^ (aromatic -CH), 1744 cm^−1^ (carbonyl stretching of carboxylate group), 1411 cm^−1^ (-CH_3_ asymmetric deformation), 1361 cm^−1^ (-CH_2_ scissoring deformation) 1033 cm^−1^ (-CO stretching), 748 cm^−1^ (NH wagging) [30]. FTIR spectra of PL depicted characteristic peaks at 3428 cm^−1^ (O-H stretching), 2916 cm^−1^ (CH_2_ stretching), 2847 cm^−1^ (CH_2_ stretching), 1788 cm^−1^ (C = O vibration), 1214 cm^−1^ (PO_2_ stretching), 839 cm^−1^ (P-O stretching). The OH stretching vibration of ETO at 3341 cm^−1^ shows a decrease in intensity for the ETO–PLC 4:6 ratio Figure 3(IIIc), indicating the involvement of this group in the complexation reaction. Furthermore, the OH peak shows a significant broadening and a decrease in intensity, and shifts to 3369 cm^−1^ for the ETO–PLC 1:1 complex Figure 3(IIId). This signifies the involvement of the OH group in complex formation [31]. The broadening of the OH peak in 1:1 ETO–PLC is more compared to that of the 4:6 ratio, indicating more hydrogen bonding and better complexation at the 1:1 ratio. The potential involvement of the OH group in complex formation also corroborates with docking studies.

Further, the marked peak shift in 1:1 ETO–PLC spectra as compared to 4:6 ETO–PLC validates the findings of the Job plot, confirming 1:1 as the optimum stoichiometric ratio for the development of ETO–PLC.

### 3.5. XRD Studies

Figure 3(II) shows diffractograms of plain ETO (Figure 3(IIa)), ETO–PLC 4:6 ratio (Figure 3(IIb)), ETO–PLC 1:1 ratio (Figure 3(IIc)). ETO shows characteristic sharp peaks at 9.39° and 18.84° 2θ, showing its crystalline nature. These peaks are similar to those reported in the literature [30].

The 4:6 complex Figure 3(IIb) exhibited a trend towards amorphization, while the diffractogram 1:1 complex Figure 3(IIc) revealed complete amorphization of the drug by the absence of any discrete sharp peak. The conversion of crystalline drug to an amorphous nature indicates complex formation [7]. The XRD studies corroborate with the results of the Job plot, which predicted maximum complexation at a stoichiometric ratio of 1:1.

### 3.6. DSC Studies

Figure 3III shows the DSC thermogram of ETO and ETO–PLC 1:1 ratio. ETO exhibited a sharp endothermic peak at 148 °C, corresponding to its melting point. These findings are in agreement with the literature-reported value [32]. This peak broadens and shifts to 134 °C in the thermogram of the complex. This conversion indicates a shift from crystalline to amorphous nature due to complex formation [33]. The results of XRD are in agreement with the findings of DSC.

### 3.7. SEM Studies

**Figure 4:**
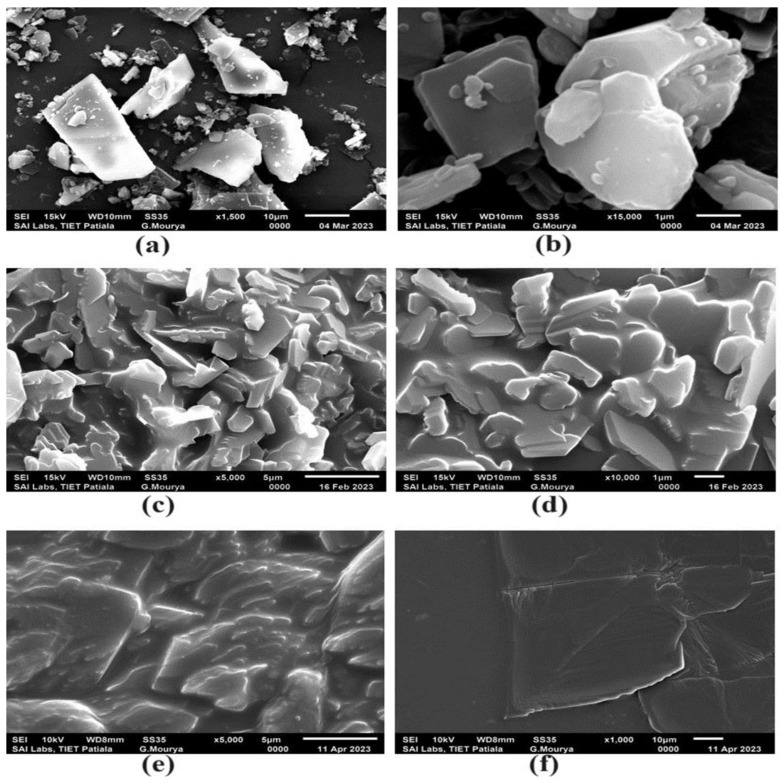
(I) SEM images at different magnifications of ETO (**a**,**b**), ETO–PLC 4:6 ratio (**c**,**d**) and ETO–PLC 1:1 ratio (**e**,**f**).

Figure 4 depicts the surface morphology of ETO, ETO–PLC 4:6, and ETO–PLC 1:1. SEM images of ETO showed a sharp-edged, flat surface, and a regular crystalline structure. The SEM images of the ETO–PLC 4:6 ratio demonstrate a reduction in the sharp edges of the drug, suggesting a reduction in crystallinity. The surface of the ETO–PLC 1:1 ratio shows marked change concerning the surface texture, with smooth and round edges, indicating complete amorphization of ETO by complex formation. The SEM results comply with XRD findings, which had predicted the amorphous nature of the complex [7].

### 3.8. ^1^H NMR Studies

**Figure 5:**
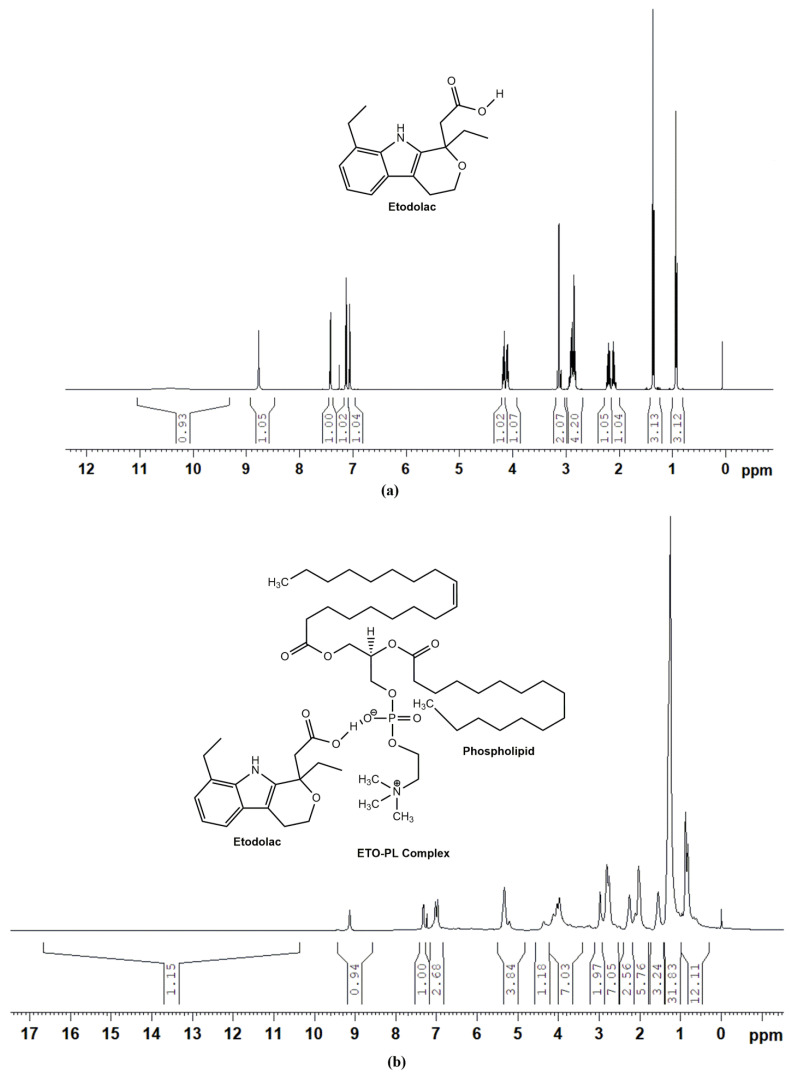
^1^H NMR spectra of ETO (**a**), ETO–PLC 1:1 (**b**).

Figure 5 shows the ^1^H NMR spectra of the plain drug (ETO) (Figure 5a) along that of ETO PLC 1:1 (Figure 5b) (Supplementary Materials). ETO showed distinct signals at δ values of 10.4725 ppm (bs, 1H, -COOH), 8.7704 ppm (s, 1H, -NH), 7.4306 ppm (d, 1H, ArH, *J* = 7.5), 7.1309 ppm (t, 1H, ArH, *J* = 7.3), 7.0648 ppm (d, 1H, ArH, *J* = 6.5), 4.1949 ppm (m, 2H, -CH_2_), 3.1689 ppm (m, 2H, -CH_2_), 2.9511 ppm (m, 4H, 2-CH_2_), 2.2301 ppm (m, 2H, -CH_2_), 1.3822 ppm (t, 3H, -CH_3_, *J* = 7.6), and 0.9510 ppm (t, 3H, -CH_3_, *J* = 7.3). The spectrum corroborates well with that reported in the literature, which confirms the purity of the ETO drug [34]. All the expected peaks for the ETO–PLC complex (1:1) are observed in the spectra. A peak shift was observed for the COOH proton from 10.4725 ppm (ETO) to 13.9407 ppm in the spectrum of the ETO–PLC (1:1) (Figure 5b). This suggested a hydrogen bonding between ETO and PL, thus confirming the desired formation of the phospholipid complex. These ^1^H NMR results are consistent with the XRD and FTIR studies reported in the study.

### 3.9. QbD Approach

#### 3.9.1. Risk Analysis

**Figure 6:**
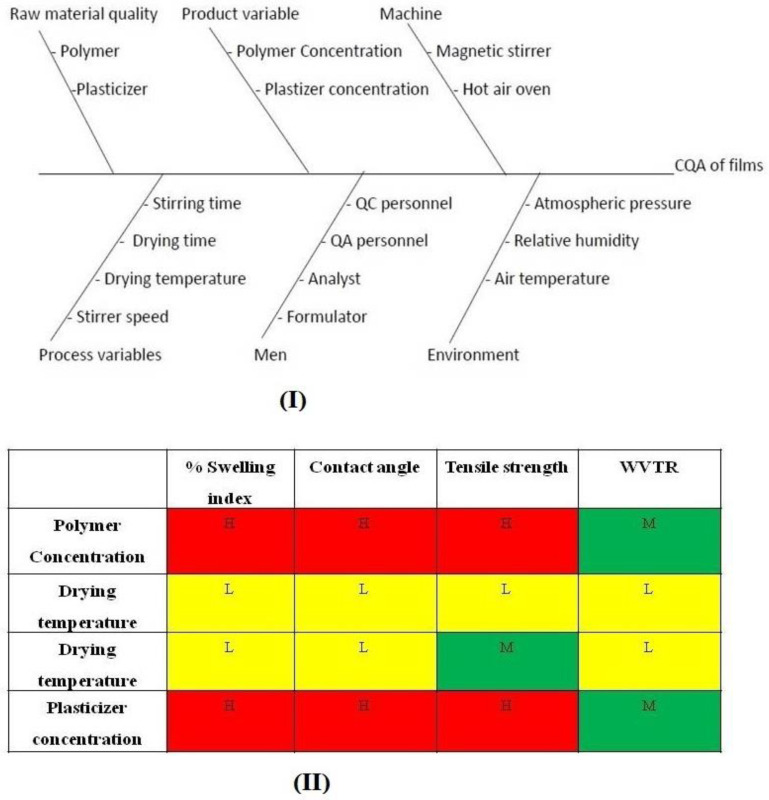
(**I**) Ishikawa fishbone diagram depicting factors affecting the critical quality attributes of films; (**II**) Risk assessment matrix (Red: high risk, yellow: medium risk and green: low risk) for the different CMAs and CPPs affecting the CQAs

CQAs affecting the film formulation were identified for development of an effective film formulation. The first criterion is that the film should be able to make good contact with the skin. This property is defined by the CA; hence, CA was selected as one of the CQAs. After making contact, the second critical factor is the uptake of moisture by the film, which would aid in polymer relaxation and promote drug release. The water uptake is indicated by SI. Furthermore, the water vapor transmission rate (WVTR) across the film influences the barrier, hydration, and protective properties of the skin. Furthermore, the mechanical strength of the film also affects film handling. Therefore SI, WVTR, and TS were selected as the CQAs. Thereafter, risk assessment was carried out using a cause-effect diagram (Ishikawa fishbone diagram) Figure 6(I). The selected CMAs and CPPs were associated with the potential risk that they pose towards the attainment of the desired CQAs. The critical factors were categorized as having a low, medium, or high risk (Figure 6(II)). From the risk assessment matrix, it can be seen that drying time and temperature were categorized as low-risk factors as these can be carefully controlled during preparation of films, and hence were not considered as critical variables. Polymer and plasticizer concentration can affect almost all the identified CQAs. A change in concentration of polymer or plasticizer can lead to alteration in hydrophilicity of the films, which would in turn lead to changes in the CQAs. Hence, these two factors were designated as the CMAs.

#### 3.9.2. Central Composite Design

Following the identification of CQAs and CMAs, a central composite consisting of 10 runs (with center point replicate) was applied. The design matrix is depicted in Table 1. Preliminary evaluation of polymeric films revealed the weight of films to be in the range of 5.55 ± 0.18 to 44.32 ± 0.04 mg and the thickness in the range of 0.13 ± 0.01 to 0.23 ± 0.07 mm. All films were folded >300 times, indicating good folding endurance of the developed films. The pH of the film was in the range of 6.8 ± 0.03, suggesting the non-irritant character to skin. The experimental results obtained for SI, CA, TS, and WVTR were analyzed using JMP^®^ student subscription 17.1.0 software. The data were fitted into various mathematical models, and the model demonstrating the highest prediction power and significant lack of fit (*p* < 0.05) was selected to be the best-fit model. Model parameters indicating goodness of fit for the different responses are shown in Table 2.

The polynomial Equations (5)–(8) describing each response as a function of input variables are as follows.

(5) $$CA=33.00\;-\;11.98X_{1}\;-\;6.36X_{2}+0.075X_{1}X_{2}+2.78X_{1}^{2}+1.34X_{2}^{2}$$

(6) $$SI=60.72+18.06X_{1}+8.67X_{2}+8.67X_{2}+2.05X_{1}X_{2}+2.52X_{1}^{2}\;-\;3.42X_{2}^{2}$$

(7) $$WVTR=15.55+5.61X_{1}+2.05\;X_{2}+1.49X_{1}X_{2}+1.23X_{1}^{2}+0.18\;X_{2}^{2}$$

(8) $$TS=2.44+0.40X_{1}+0.028X_{2}+0.02X_{1}X_{2}\;-\;0.21X_{1}^{2}\;-\;0.182X_{2}^{2}$$

**Figure 7:**
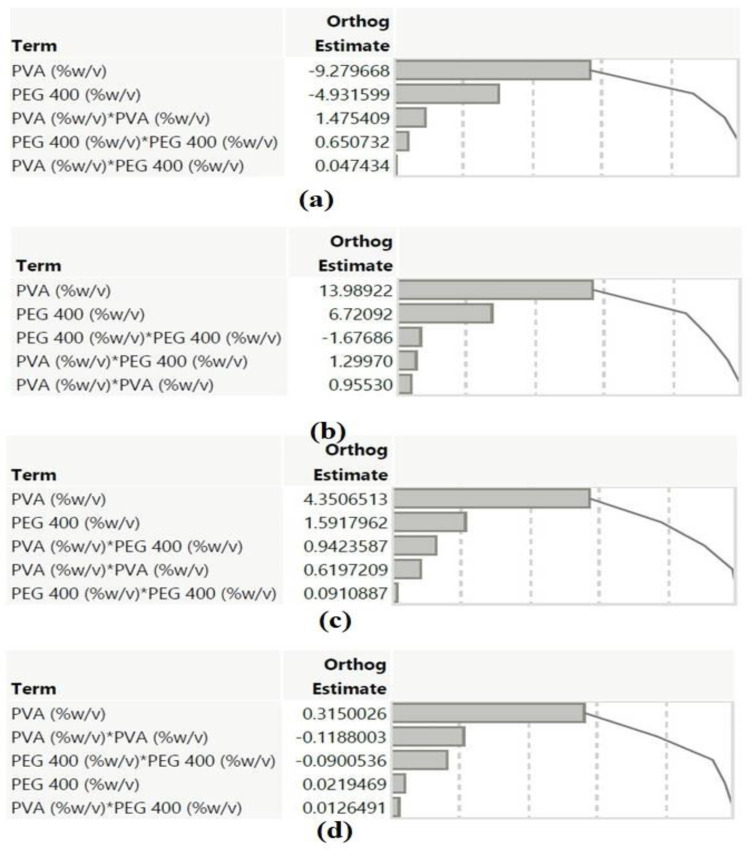
Pareto plots of contact angle (**a**), swelling index (**b**), water vapour transmission rate (**c**), and tensile strength (**d**).

**Figure 8:**
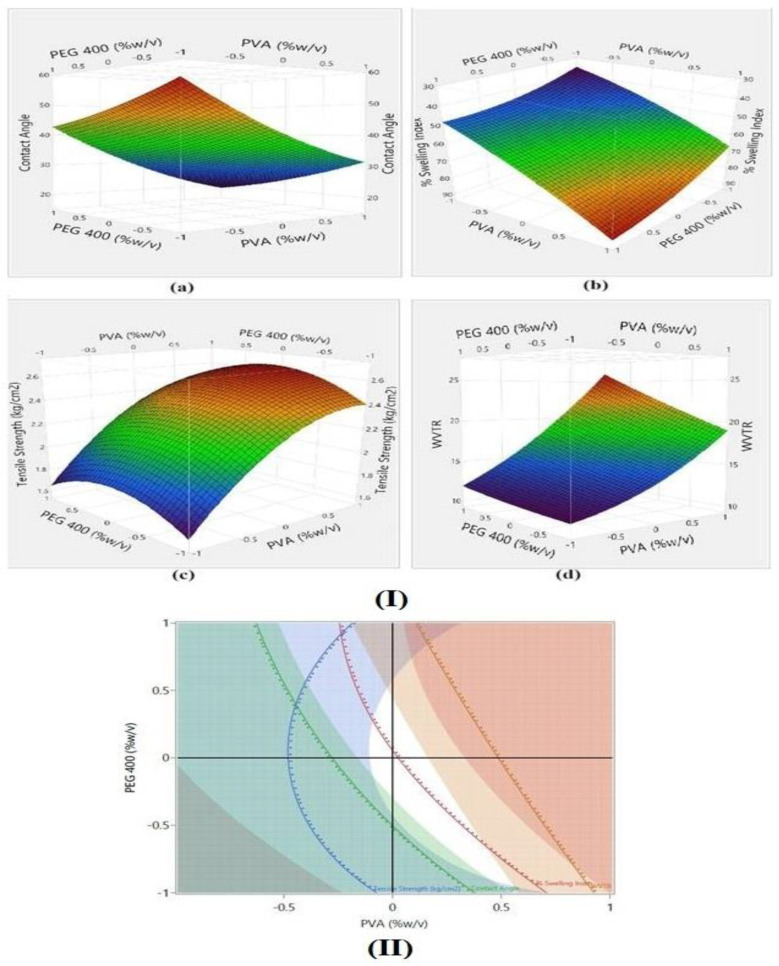
(**I**) Response surface plots for CA (**a**), swelling index (**b**), tensile strength (**c**), and water vapour transmission rate (**d**); (**II**) Desirability plot and the optimized design space (white indicates the desirability region, other colors indicate the region for other CQAs).

The model evaluation parameters for CA indicate a negative relationship between CA and both polymer and plasticizer concentration, with X_1_ (PVA concentration) having a profound effect on CA in comparison to X_2_ (PEG concentration). The same is reflected in the Pareto plot showing the effect of factors on CA Figure 7a. This is due to the fact that CA is also dependent on the hydrophilicity of the films. As the concentration of hydrophilic polymer increases, the hydrophilicity increases and, consequently, the CA decreases, leading to better contact and wetting of the film at the application site. The response surface plot shows a curvilinear trend for CA (Figure 8(Ia)). From the model-fitting parameters, it is clear that both X_1_ and X_2_ have a positive influence on SI. The response surface plots (Figure 8(Ib)) show a sharp ascending trend in SI as the PVA concentration increases. A rising trend is also observed with increased PEG 400 concentration, though it is less pronounced than the effect of PVA. This is also evident in the Pareto plots (Figure 7b). As the amount of hydrophilic polymer or plasticizer increases, the ability of the film to take up water and swell also increases. At higher PEG 400 and PVA concentrations, excessive swelling was observed.

The model evaluation parameters for TS and WVTR also indicate a positive effect of X_1_ and X_2_ on both responses. The model estimates in terms of Pareto plots are depicted in Figure 7, where the effect of X_1_ is more pronounced than that of X_2_, while the interaction terms have less effect. The mechanical strength improves with an increase in the polymeric structure of the film and hence, increased polymer concentration has a positive influence on TS. The WVTR is also a function of increased chain relaxation and hydrophilicity, so increased polymer and plasticizer content led to an increase in WVTR. The response surface plots are shown in Figure 8.

#### 3.9.3. Model Validation

Numerical optimization was carried out to maximize desirability. Figure 8(II) shows the obtained design space for developing an optimized formulation. From the obtained design space, PEG400 and PVA, each at coded level 0, were selected as optimized formulations, as well as for predicting model validity. The observed values for SI, CA, TS, and WVTR were 60.14 ± 1,01%. 31.6 ± 0.03. 2.44 ± 0.39 kg/cm^2^, and 15.38 g/hm^2^, respectively. These values were close to the model-predicted values with good correlation, proving the validity of the developed model. This selected film (F6) was then loaded with drug–phospholipid complex (ETO–PLC-F6) and was subjected to further studies.

### 3.10. In Vitro Drug Diffusion Studies

**Figure 9:**
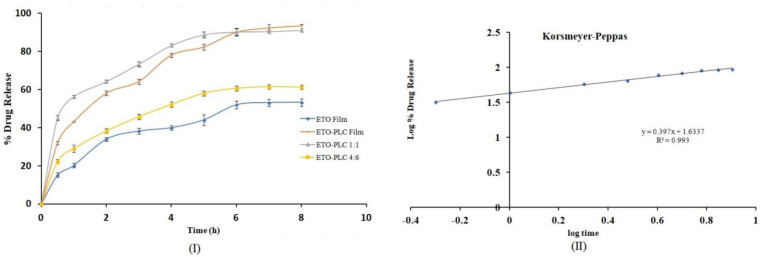
(**I**) In vitro drug diffusion profile of of ETO–PLC and ETO–PLC loaded films; (**II**) ETO–PLC loaded films release data fitting into the Korsmeyer–Peppas equation.

In vitro diffusion of ETO, ETO–PLC 1:1, ETO–PLC 4:6, and optimized ETO–PLC-loaded transdermal films is shown in Figure 9(I). The ETO-containing films and ETO–PLC 4:6 complex demonstrated incomplete release of 53% and 61%, respectively, at the end of 8 h. The drug release improved with ETO–PLC 1:1 complex to 91.09 ± 0.92% over a period of 8 h. The higher release can be attributed to the better dissolution and solubility characteristics of the complex. However, 1:1 complex showed initial burst release while the films showed a sustained release of 93.40 ± 0.99% over a period of 8 h. Furthermore, the drug release data of optimized ETO–PLC F6 film were fitted into various rate equations to estimate the rate of drug release. The R^2^ value obtained for data fitting into zero order, first order, Hixon Crowell, and Higuchi were 0.9232, 0.9883, 0.9232, and 0.9821, respectively. However, the data showed the best fit to the Korsmeyer–Peppas equation, with R^2^ of 0.993 (Figure 9(II)). Furthermore, the release exponent was calculated as 0.397, indicating that ETO release from the film formulation is governed by fiction diffusion.

### 3.11. Permeation Studies

Permeation studies were conducted on ETO–PLC film and ETO films. It was found that the cumulative amount of ETO from ETO–PLC films was significantly (*p* < 0.05) higher than the amount permeated from ETO samples. Furthermore, the former demonstrated a higher flux (2.61 ± 0.001 mg/cm^2^/h) value as compared to the latter (1.94 ± 0.002 mg/cm^2^/h). It was found that there was a direct relationship between steady-state flux and permeability coefficients. The permeability coefficient of ETO–PLC film (0.008 ± 0.015 cm/h) was also significantly (*t*-test; *p* < 0.05) higher as compared to ETO films (0.006 ± 0.025 cm/h). This can be attributed to the improved solubility and permeability characteristics after phospholipid complexation.

### 3.12. In Vivo Anti-Inflammatory Activity

The rats were subjected to a paw edema test to check the anti-inflammatory activity using the carrageenan paw edema model. Carrageenan induces inflammation in two phases. The initial phase involves inflammation by vasodilation accompanied by the release of inflammatory markers like histamine, 5–HT, and bradykinin. The second phase involves the release of TNF–α and infiltration of macrophages, eosinophils, and lymphocytes. The diseased group showed significant augmentation (*p* < 0.05) in edema when compared to the control group. A significant attenuation (*p* < 0.05) in paw edema was obtained for the treatment group II, in comparison to the disease group and treatment I group, demonstrating the improved anti-inflammatory activity of the developed formulation.

## 4. Conclusions

In the present study, ETO–PLC was prepared by a solvent evaporation method. The complex formation was confirmed by H NMR, DSC, XRD, and SEM studies. The prepared complex was further incorporated in films using methyl cellulose and polyvinyl alcohol as polymers and PEG 400 as plasticizers. Films were optimized using the QbD approach to obtain the optimized design space using the desirability approach. Polymer and plasticizer concentration were the independent variables, while CA, WVTR, TS, and SI were the dependent variables. The optimized formulation exhibited sustained drug release and improved permeation across the skin. In vivo anti-inflammatory studies also confirmed the improved anti-inflammatory effect of the developed formulation. Overall, the development of etodolac–phospholipid complex-loaded polymeric films is an effective strategy for improving the biopharmaceutical profile of etodolac.

## Figures and Tables

**Table 1:** Design matrix for optimization of blank films.

Independent Variable	Type	Level		
		Low (−1)	Medium (0)	High (+1)
PVA concentration (X1) (% *w*/*v*)	Continuous	2	3	4
PEG 400 concentration (X2) (% *w*/*v*)	Continuous	5	6.5	8
Optimization Design matrix with 10 trials *				
Trial	X1		X2	
F1	1		1	
F2	−1		0	
F3	0		0	
F4	0		−1	
F5	−1		−1	
F6	0		0	
F7	1		−1	
F8	1		0	
F9	−1		1	
F10	0		1	

* All formulations contained 2% *w*/*v* methylcellulose.

**Table 2:** Model prediction parameters for various CQAs.

Response	RMSE	R^2^	*p*-Value
CA	3.556	0.96	0.0077
SI	3.0732	0.98489	0.0010
WVTR	0.9246	0.98519	0.0009
TS	0.0991	0.97	0.0041

## Data Availability

The original contributions presented in the study are included in the article, further inquiries can be directed to the corresponding author/s.

## Supplementary Materials

The following supporting information can be downloaded at: https://www.mdpi.com/article/10.3390/polym16172517/s1, Figure S1: Supplementary NMR spectra file.
