# Supplementary material for: A QbD-Navigated Approach to the Development and Evaluation of Etodolac–Phospholipid Complex Containing Polymeric Films for Improved Anti-Inflammatory Effect
Source: Polymers (Basel). 2024 Sep 4;16(17):2517. doi: 10.3390/polym16172517 (PMC11398249; doi:10.3390/polym16172517)
Supplement: Supplementary file 1 [file polymers-16-02517-s001.zip › polymers-3163482-supplementary.pdf]

2

1H_8scan CDCl3 {D:\Spectra} nmr 21

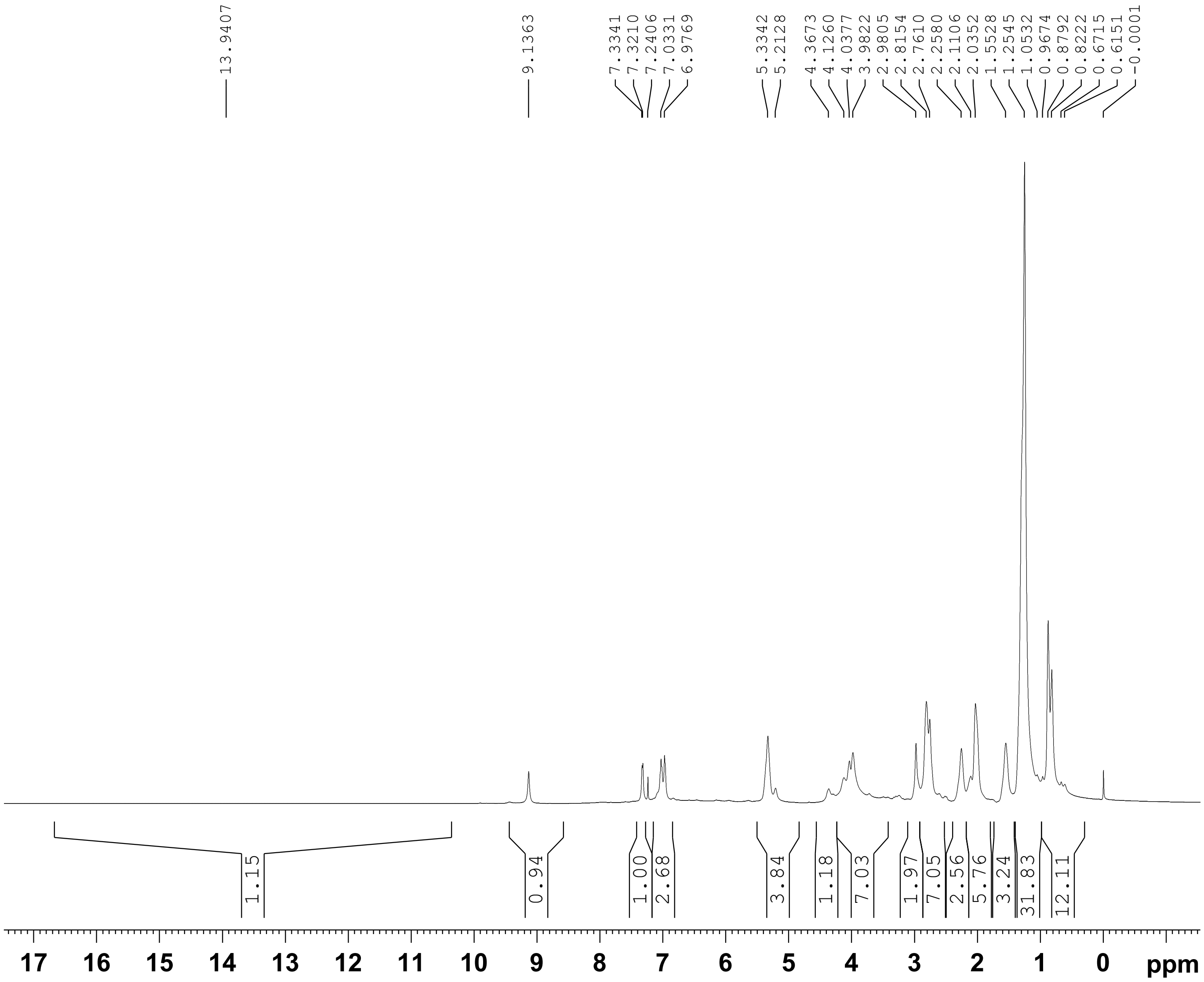

BRUKER
AVANCE NEO
500 MHz NMR
SPECTROMETER
SAIF, P.U.

Current Data Parameters

NAME	May08-2023
EXPNO	210
PROCNO	1

F2 - Acquisition Parameters

Date_	20230508
Time	16.07 h
INSTRUM	Avance Neo 500
PROBHD	Z119470_0333 (
PULPROG	zg30
TD	65536
SOLVENT	CDCl3
NS	32
DS	0
SWH	14705.883 Hz
FIDRES	0.448788 Hz
AQ	2.2282240 sec
RG	22.1362
DW	34.000 usec
DE	6.79 usec
TE	300.1 K
D1	1.00000000 sec
TD0	1
SFO1	500.1730885 MHz
NUC1	1H
P0	3.33 usec
P1	10.00 usec
PLW1	20.93000031 W

F2 - Processing parameters

SI	65536
SF	500.1700211 MHz
WDW	EM
SSB	0
LB	0.30 Hz
GB	0
PC	1.00

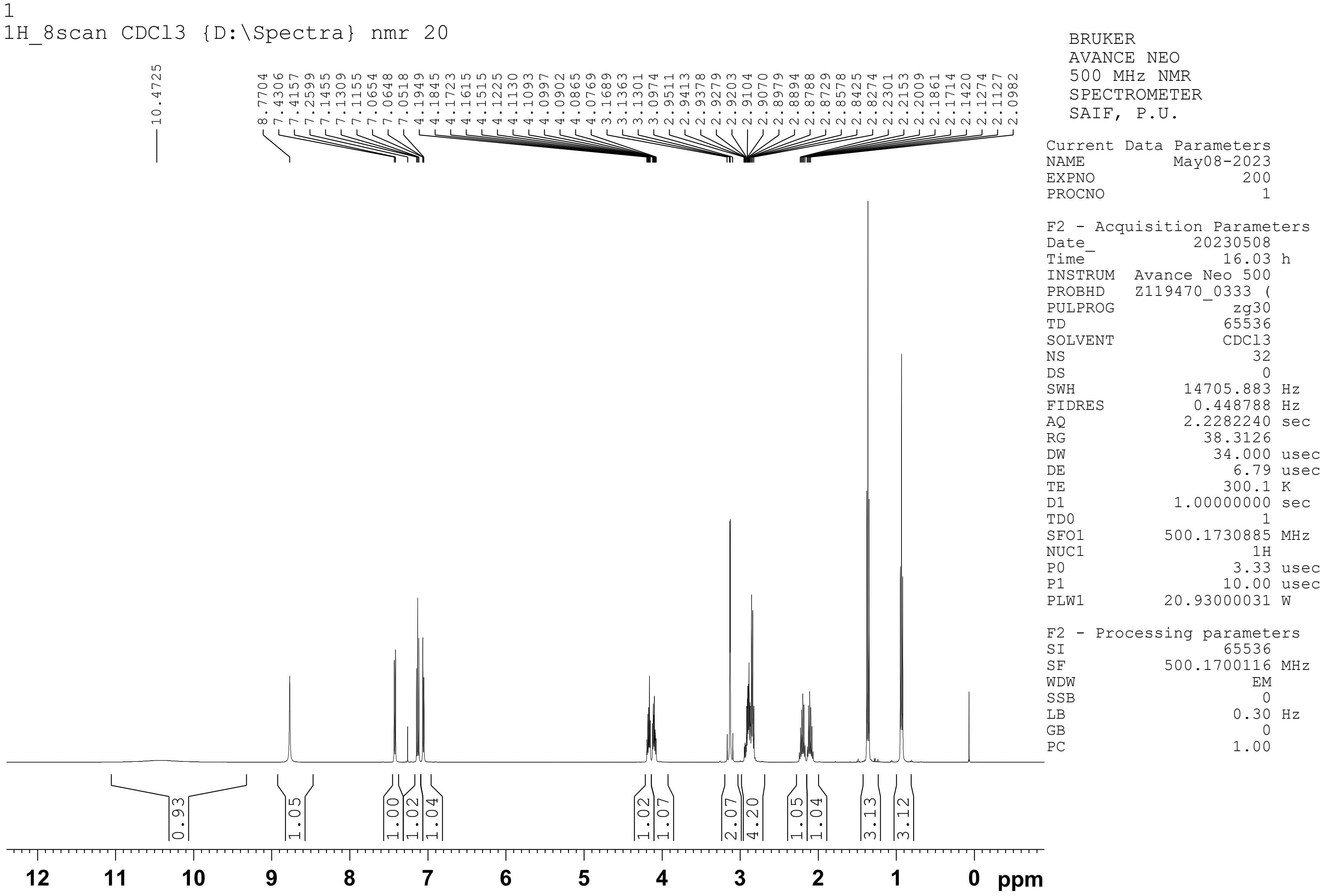

Figure S1. Supplementary NMR spectra file.
